# Proteasomes generate spliced epitopes by two different mechanisms and as efficiently as non-spliced epitopes

**DOI:** 10.1038/srep24032

**Published:** 2016-04-06

**Authors:** F. Ebstein, K. Textoris-Taube, C. Keller, R. Golnik, N. Vigneron, B. J. Van den Eynde, B. Schuler-Thurner, D. Schadendorf, F. K. M. Lorenz, W. Uckert, S. Urban, A. Lehmann, N. Albrecht-Koepke, K. Janek, P. Henklein, A. Niewienda, P. M. Kloetzel, M. Mishto

**Affiliations:** 1 Institut für Biochemie, Charité-Universitätsmedizin Berlin, Charitéplatz 1, D-10117 Berlin, Germany; 2 Ludwig Institute for Cancer Research, WELBIO (Walloon Excellence in Life Sciences and Biotechnology) and the de Duve Institute, Université catholique de Louvain, Place de l’Université 1, B-1200 Brussels, Belgium; 3 Department of Dermatology, Universitätsklinikum Erlangen, Ulmenweg 18, D-91052 Erlangen, Germany; 4 Klinik für Dermatologie, Venerologie und Allergologie, Universitätsklinikum Essen D - 45122 Essen, Germany & German Cancer Consortium (DKTK), Hufelandstraße 55, D-69120 Heidelberg, Germany; 5 Max-Delbrück-Center for Molecular Medicine, Robert-Rössle-Str. 10, D-13092 Berlin, Germany; 6 Institute of Biology, Humboldt University Berlin, Charitéplatz 1, D-10115 Berlin, Germany

## Abstract

Proteasome-catalyzed peptide splicing represents an additional catalytic activity of proteasomes contributing to the pool of MHC-class I-presented epitopes. We here biochemically and functionally characterized a new melanoma gp100 derived spliced epitope. We demonstrate that the gp100^mel^_47–52/40–42_ antigenic peptide is generated *in vitro* and *in cellulo* by a not yet described proteasomal condensation reaction. gp100^mel^_47–52/40–42_ generation is enhanced in the presence of the β5i/LMP7 proteasome-subunit and elicits a peptide-specific CD8^+^ T cell response. Importantly, we demonstrate that different gp100^mel^-derived spliced epitopes are generated and presented to CD8^+^ T cells with efficacies comparable to non-spliced canonical tumor epitopes and that gp100^mel^-derived spliced epitopes trigger activation of CD8^+^ T cells found in peripheral blood of half of the melanoma patients tested. Our data suggest that both transpeptidation and condensation reactions contribute to the frequent generation of spliced epitopes also *in vivo* and that their immune relevance may be comparable to non-spliced epitopes.

CD8^+^ T cell responses involve in most cases the proteasome-dependent processing of antigens and cell surface presentation of the resulting peptides by MHC-class I molecules to cytotoxic T lymphocytes (CTLs). The 20S standard proteasome (s-proteasome) with its active site β-subunits β1, β2 and β5, the immunoproteasome (i-proteasome) with the IFN-γ-induced catalytic β-subunits β1i/LMP2, β2i/MECL1 and β5i/LMP7 or intermediate proteasome types containing both standard and immuno-subunits^1^,^2^ are the catalytic cores of 30S proteasomes, which are formed by the association of two 19S regulator complexes with the 20S core^3^. The 30S proteasome isoforms execute the regulated degradation of ubiquitin (Ub)-tagged proteins, thereby generating peptides presented by MHC-class I proteins^4^,^5^. Exchange of standard catalytic subunits for the corresponding IFN-γ-induced β-subunits leads to variations of proteasome catalytic pocket structure and of the peptide transport along the proteasome inner channel^6^,^7^,^8^. Such variations may result in quantitative differences in the proteasome cleavage products, which in turn can strongly affect cell surface presentation of MHC-class I-bound virus- or tumor-specific antigenic peptides and in consequence also the efficacy of a peptide-specific CD8^+^ T cell response^9^,^10^.

Canonical MHC-class I-bound peptides generated by proteasomes mirror the linear sequence of the parental protein^10^. In contrast, spliced peptides that are presented by MHC-class I molecules and generated by proteasome-catalyzed peptide splicing (PCPS) result from the ligation of two separate distant proteasomal cleavage products that are not contiguous in the parental protein. We previously provided experimental proofs for the hypothesis that PCPS proceeds via a transpeptidation reaction^11^,^12^,^13^,^14^. PCPS involves the formation of an *O*-acyl-enzyme intermediate consisting of a N-terminal peptide fragment linked to the Thr1 residue of one of the β-subunit active sites. In the course of the transpeptidation reaction, this acyl-enzyme intermediate is subjected to a nucleophilic attack by the amino-terminus of another peptide fragment leading to the creation of a new peptide containing two non-contiguous fragments of a protein^11^,^12^,^15^. Theoretically, spliced peptides may also be produced without the formation of a semi-stable *O*-acyl-enzyme intermediate by a so-called condensation reaction. Condensation reactions forming *de novo* peptide bonds have been described for some proteases to occur *in vitro* under conditions disfavoring diffusion of peptides from the active sites^16^,^17^. However, whether such a condensation reaction can take place *in cellulo* in the barrel-shaped proteasome controlling peptide diffusion and whether this can contribute to epitope production has yet to be shown.

Five spliced epitopes, derived from the fibroblast growth factor 5 (FGF-5_172–176/217–220_), melanocyte protein gp100^mel^ (gp100^mel^_40–42/47–52_; gp100^mel^_195–202/192_), the SP100 nuclear phosphoprotein (SP100_296–301/286–289_) and tyrosinase (Tyr_368–373/336–340_) have been identified so far using cancer patient-derived CTLs for epitope identification^11^,^12^,^18^,^19^,^20^. Interestingly, two of these spliced peptide-specific CD8^+^ T cells have previously been shown to induce regression of tumors in a clinical setting^20^,^21^ or the engraftment of acute myeloid leukemia cells in non-obese diabetic/SCID mice^11^,^22^, indicating their potential immune relevance.

PCPS represents a genuine catalytic activity of proteasomes competing with the normal proteasomal hydrolysis event^14^. Initial estimations of epitope splicing efficacy were calculated to range between 0.0002% and 0.01% of the total proteasome-dependent epitope generation. Therefore, epitope production by PCPS was thought to be an extremely rare event^12^,^13^,^16^. However, performing *in vitro* experiments we showed that PCPS may be a relatively frequent process controlled by certain sequence requirements guaranteeing that PCPS is also a highly reproducible reaction^14^. In fact, *in vitro* experiments suggested that potential MHC-class I epitopes are relatively more frequent among spliced peptides than among the “canonical” non-spliced proteasome cleavage products^14^,^23^. The limited number of spliced tumor epitopes identified most likely resides in the fact that identification of immune-relevant spliced peptides has so far been limited by the serendipitous availability of patient-derived CTLs and the concomitant considerable experimental effort to identify the cognate epitopes.

To close this gap, we here report an algorithm-aided reverse immunology approach permitting the facilitated identification of spliced peptides from a given protein sequence thereby overcoming the need for patient-derived CTLs. By applying this method we here identified a new spliced peptide derived from melanocytic protein gp100^mel^. This peptide can be generated *in vitro* and *in cellulo* by a condensation reaction. This epitope is presented by patient-derived melanoma cells and triggers activation of CD8^+^ T cells found in the peripheral blood of melanoma patients. Importantly, we show that spliced epitopes are presented at the cell surface and trigger CTL responses with efficacies similar to non-spliced epitope underlining that PCPS may play an important role in anti-tumor immune responses.

## Results

### The gp100^mel^_47–52/40–42_ antigenic peptide is generated by a new proteasomal catalytic mechanism

**Figure 1:**
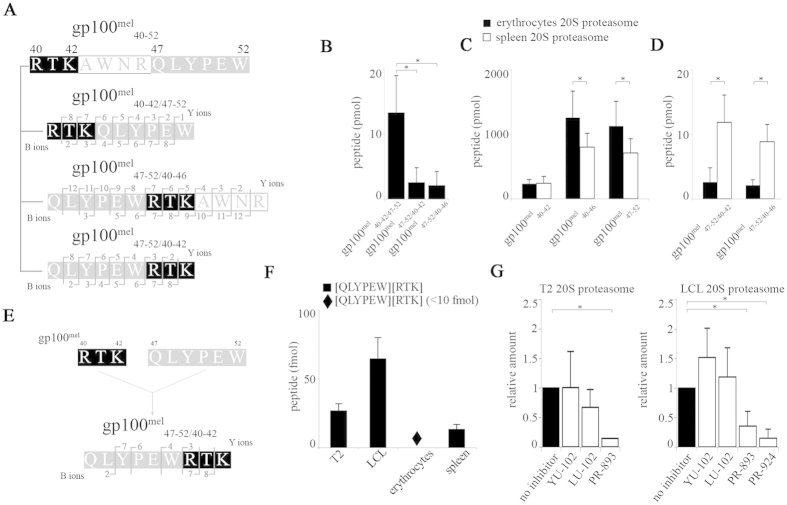
Involvement of proteasome standard- and immuno-subunits in *in vitro* spliced peptide generation. (**A**) B- and Y-ions of the spliced products gp100^mel^_40–42/47–52_ [RTK][QLYPEW], gp100^mel^_47–52/40–42_ [QLYPEW][RTK] and gp100^mel^_47–52/40–46_ [QLYPEW][RTKAWNR] as detected by MS/MS Orbitrap analysis (see also Fig. S1) and derived from gp100^mel^_40–52_ upon 20S proteasome-catalyzed digestion. (**B**) Erythrocyte 20S s-proteasomes generated larger amount of the spliced peptide gp100^mel^_40–42/47–52_ than of the gp100^mel^_47–52/40–42_ and gp100^mel^_47–52/40–46_ peptides. (**C,D**) Erythrocyte 20S s-proteasomes generated similar or larger amounts of the splice reactants gp100^mel^_40–42_ [RTK], gp100^mel^_40–46_ [RTKAWNR] and gp100^mel^_47–52_ [QLYPEW] (**C**) but less gp100^mel^_47–52/40–42_ and gp100^mel^_47–52/40–46_ spliced peptides than spleen 20S proteasomes (**D**). (**B–D**) The means of the amount measured after 3 hrs *in vitro* digestion and the SD between triplicates of two independent experiments are shown. Significant differences of the means are detected by student t test and marked by *. In particular, (**B**) gp100^mel^: _40–42/47–52_ *vs*_47–52/40–42_, p = 0.002; _40–42/47–52_ *vs*_47–52/__40–46_, p < 0.001; (**C**) gp100^mel^_40–46_ s- *vs* i-proteasome, p = 0.036; gp100^mel^_47–52_ s- *vs* i-proteasome, p = 0.043; (**D**) gp100^mel^_47–52/40–42_: s- *vs* i-proteasome p < 0.001; gp100^mel^_47–52/40–46_: s- *vs* i-proteasome p < 0.001. (**E)** B- and Y-ions of peptide gp100^mel^_47–52/40–52_ -detected by MALDI-TOF/TOF-MS (Fig. S3) – that was produced by hydrolysis-independent ligation of the splice-reactants [RTK] and [QLYPEW] by 20S proteasome. (**F**) The peptide gp100^mel^_47–52/40–42_ amount, generated by hydrolysis-independent ligation by i- and s-proteasomes is shown. Amounts below 10 fmol could be detected but not quantified and are symbolized with ♦. (**G**) Absolute amounts of gp100^mel^_47–52/40–42_ generated in the hydrolysis-independent assay by T2 and LCL 20S proteasomes in the presence of the inhibitors YU-102 (2 μM; β1/β1i-specific), LU-102 (2 μM; β2/β2i-specific), PR-893 (0.5 μM; β5-specific) and PR-924 (0.5 μM; β5i-specific). Significant differences due to the effect of the inhibitors are detected by paired student t test and marked by * (T2 proteasome, control *vs* PR-893, p = 0.037; LCL proteasome, control *vs* PR-893, p = 0.050; control *vs* PR-924, p = 0.012). (**F,G)** The mean (fmol measured in 9 μl reaction) and the SD of independent experiments (n = 2–3), each measured in two duplicates are shown.

For identification of new spliced peptides and/or epitopes we subjected the gp100^mel^-derived synthetic polypeptide substrate gp100^mel^_40–52_ [RTKAWNRQLYPEW] to *in vitro* digestion by purified human 20S proteasomes. By applying SpliceMet^23^ we identified three spliced peptides among the proteasomal processing products. These were the previously reported spliced antigenic peptide gp100^mel^_40–42/47–52_ [RTK][QLYPEW]^12^ and the novel gp100^mel^_47–52/40–46_ [QLYPEW][RTKAWNR] and gp100_47–52/40–42_ [QLYPEW][RTK] peptides (Fig. 1A, Fig. S1). Interestingly, a theoretical IC_50_ value of 86.1 μM for HLA-A*03:01 and HLA-A*011 binding was predicted for gp100^mel^_47–52/40–42_ [QLYPEW][RTK] by *in silico* analysis^24^. Therefore, we decided to analyze the formation of this potential new spliced tumor epitope in more detail.

Monitoring the *in vitro* generation of the spliced peptides revealed that the spliced peptide gp100^mel^_40–42/47–52_ was produced approximately 6-fold more efficiently by erythrocytes s-proteasomes than the new spliced gp100^mel^_47–52/40–42_ peptide, being composed of the same peptide fragments but in reversed order (Fig. 1B). Interestingly, opposite to the generation kinetics of the individual splice-reactants [QLYPEW], [RTKAWNR] or [RTK] (Fig. 1C), spleen i-proteasomes generated the spliced peptides gp100_47–52/40–46_ and gp100_47–52/40–42_ more efficiently than erythrocytes s-proteasomes (Fig. 1D, Fig. S2).

Both the gp100^mel^_47–52/40–42_ and gp100^mel^_47–52/40–46_ peptides were the result of the linkage between the gp100 Trp_52_ and Arg_40_ residues of the gp100-derived substrate (Fig. 1E). However, the Trp_52_ residue of the N-terminal splice-reactant [QLYPEW] represents the C-terminal residue of the synthetic polypeptide substrate gp100^mel^_40–52_. This excluded the formation of an *O*-acyl-enzyme intermediate with the proteasomal active site Thr_1_ as a result of peptide-bond hydrolysis as demanded for the established transpeptidation reactions^12^,^14^. We therefore hypothesized that spliced peptides could also be produced in a proteasome-catalyzed condensation reaction.

To test this hypothesis we co-incubated purified 20S proteasomes with the two splice-reactant peptides gp100^mel^_40–42_ [RTK] and gp100^mel^_47–52_ [QLYPEW]. The resulting spliced peptide products were then analyzed by mass spectrometry. In support of our hypothesis, 20S proteasomes catalyzed the formation of the gp100^mel^_47–52/40–42_ peptide *in vitro* when the two peptides were added individually (Fig. 1E, Fig. S3). In accordance with the results shown in Fig. 1D i-proteasomes derived from different cellular sources catalyzed the condensation reaction with higher efficiency than s-proteasomes (Fig. 1F).

In principle, all active sites of both proteasome isoforms are capable of catalyzing a transpeptidation reaction^14^. However, because condensation reactions require specific favorable conditions^16^ we next asked whether any of the proteasome active sites were specifically involved in the condensation reaction. To do so, proteasome active site subunit specific inhibitors, whose active site specificity had been previously documented^25^ were added to 20S proteasomes derived from T2 cells (s-proteasome) or LCLs (i-proteasome) together with the two splice-reactants [RTK] and [QLYPEW]. PR-893 (0.5 μM) was used to block the activity of the β5 subunits of 20S s-proteasomes and PR-924 (0.5 μM) to inhibit the activity of the β5i immuno-subunit^25^. Blockage of either the β5 active site as in T2 cell-derived 20S proteasome or the β5 and β5i active sites as present in LCL-derived proteasomes led to a strong reduction of the condensation reaction (Fig. 1G). In contrast, blocking the β1/β1i and β2/β2i active sites with YU-102 (2 μM) and LU-102 (2 μM) proteasome inhibitors^25^, respectively, barely affected the production of the antigenic peptide, suggesting that the condensation reaction was mainly executed by the active sites of the β5 subunit isoforms (Fig. 1G).

### Cellular condensation reaction results in the presentation of the gp100^mel^_47–52/40–42_ peptide

**Figure 2:**
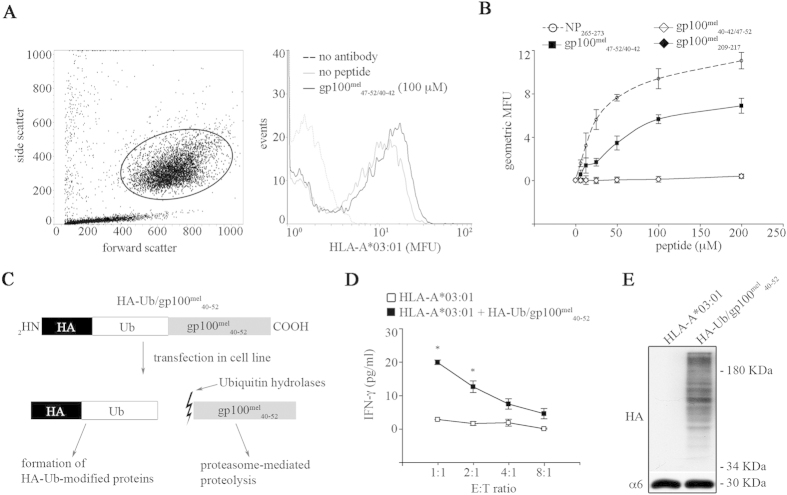
The spliced gp100^mel^_47–52/40–42_ peptide is generated from the gp100^mel^_40–42_ peptide by a condensation reaction *in cellulo*. (**A,B**) The spliced peptide gp100^mel^_47–52/40–42_ [QLYPEW][RTK] bound the HLA-A*03:01 complex on the cell surface of HeLa (**A**) and T2 (**B**) cell lines. In (**A**) the side / forward scatters charts are reported in the left panel. Cells included in the analysis are marked with a circle and their HLA-A*03:01 amount is shown in the right panel. (**B**) Shown is the binding affinity of the spliced peptide gp100^mel^_47–52/40–42_ compared to a known good HLA-A*03:01 – binder, *i.e*. [ILRGSVAHK] (NP_265–273_)^35^ and to the negative controls HLA-A*32:01-restricted gp100^mel^_40–42/47–52_ [RTK][QLYPEW] and HLA-A*02:01-restricted gp100^mel^_209–217_ [IMDQVPFSV] epitopes. Cells were transfected with HLA-A*03:01 protein encoding plasmids and pulsed with 100 μM (**A**) or different peptide concentrations (**B**). Binding affinity was measured by staining of HLA-A*03:01 and FACS analysis. Values are in mean fluorescence unit (MFU) and are means and SD of 2 replicates in a representative assay of 2–6 independent experiments. (**C**) Schematic representation of the HA-Ub/gp100^mel^_40–52_ fusion construct used in this study. The first amino acid of the gp100^mel^_40–52_ [RTKAWNRQLYPEW] sequence was directly fused in frame to the C-terminus of the Ub, which was N-terminally tagged with the YPYDVPDYA HA sequence. When expressed in cells, Ub hydrolases cleaved the fusion-polypeptide directly after the last amino acid (Gly_76_) of Ub, liberating the gp100^mel^_40–52_ peptide from the first residue. (**D**) CTL response towards HeLa 33/2 cells transfected with HLA-A*03:01 individually or together with HA-Ub/gp100^mel^_40–52_ for 24 hrs. Their ability to present gp100_47–52/40–42_ epitope was measured by using K631 1C CTL. After 16 hrs co-culture, the IFN-γ concentration in supernatants was determined by ELISA. Values are means and SD of 2 replicates in a representative assay of 6 independent experiments. Significant differences in the means of replicates are marked by * (student t-test; E:T ratio: 1:1, p = 0.003; 2:1, p = 0.050). (**E**) 10 μg of whole HeLa 33/2 cell extracts were resolved on a 15% SDS-PAGE for western blotting with anti-HA antibody to control the expression of the construct. The western blot is representative of 5 independent assays.

Although our *in vitro* experiments clearly demonstrated that proteasomes are able to generate spliced peptides in a condensation reaction, there still remained the possibility that the *in vitro* experiments as performed here may be artificially influenced by high enzyme and/or substrate concentrations. Therefore, to assess whether the gp100^mel^_47–52/40–42_ peptide is of any functional relevance and to study whether proteasomes could perform condensation reactions also *in cellulo* we established an immunological read-out system to monitor the generation and MHC class I presentation of gp100^mel^_47–52/40–42_ in HeLa cells. HLA-A binding affinity assays using HLA-A*03:01- transfected HeLa and T2 cells lines (Fig. 2A,B) as well as mature dendritic cells (data not shown) confirmed the predicted binding of the peptide to HLA-A*03:01 complex. We therefore generated a gp100^mel^_47–52/40–42_ peptide-specific CTL clone from peripheral blood mononuclear cells (PBMCs) of a HLA-A*03:01^+^ healthy donor (Fig. S4) allowing us to monitor the generation of this potential MHC class I-restricted epitope *in cellulo*.

To monitor the condensation reaction *in cellulo*, HeLa^HLA-A*03:01+^ cells were transfected with a plasmid expressing a HA-Ub-gp100^mel^_40–52_ fusion protein (Hela^A*03:01+Ub/gp100mel^_40–52_). In the fusion protein the GG motif of the ubiquitin was directly linked to the first amino acid residue of the 13-mer gp100_40–52_ peptide allowing the release of the unchanged full-length gp100^mel^_40–52_ peptide substrate after cleavage behind the GG motif by de-ubiquitinating enzymes (Fig. 2C). CTL assays using the gp100^mel^_47–52/40–42_ specific CD8^+^ T cell clone demonstrated that proteasomes could indeed also perform a condensation reaction *in cellulo* (Fig. 2D,E), indicating that in addition to transpeptidation also condensation reactions may also contribute to the cellular pool of spliced epitopes.

### The gp100^mel^_47–52/40–42_ peptide is a tumor epitope presented by melanoma cells

**Figure 3:**
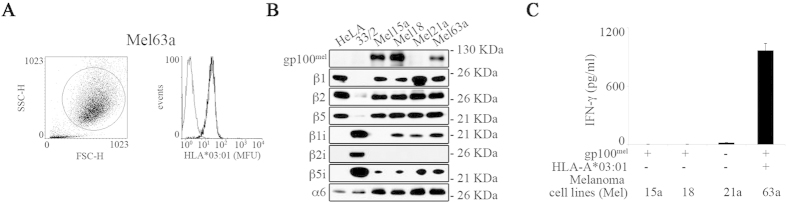
Melanoma Mel63a cells efficiently generate and present the gp100^mel^_47–52/40–42_ epitope. (**A**) Overlay histograms obtained by flow cytometry showing the cell surface expression of the HLA-A*03:01 molecule (thick line) compared to isotype control (thin line) of the Mel63a melanoma cell line. Shown is a representative staining of 2 independent experiments. (**B**) The four melanoma cell lines Mel15a, Mel18, Mel21a and Mel63a were analyzed by western blotting for their content of gp100^mel^, the proteasome subunits β1, β2, β5, β1i, β2i, β5i and α6 (the latter as loading control). HeLa cells and HeLa 33/2 cells were used as controls for s- and i-proteasomes, respectively. (**C**) The four melanoma cell lines Mel15a (HLA-A*03:01^−^/gp100^+^); Mel18 (HLA-A*03:01^−^/gp100^mel+^), Mel21a (HLA-A*03:01^−^/gp100^mel−^) and Mel63a (HLA-A*03:01^+^/gp100^mel+^) were assessed for their ability to present the gp100^mel^_47–52/40–42_ epitope in a 16 hrs CTL assay to K631 1C CTL clone. Supernatants were collected and tested for their IFN-γ content by ELISA. Values are means and SD of 2 replicates in a representative assay of 2 independent experiments.

This data raised the question whether the proteasome-catalyzed generation of gp100^mel^_47–52/40–42_ was solely determined by the biochemical nature of the 13-mer gp100^mel^_40–52_ substrate and/or the specific assay conditions applied *in vitro* and/or *in cellulo*. To further validate our reverse immunology approach, we next investigated whether the gp100^mel^_47–52/40–42_ peptide was also naturally produced from full-length gp100^mel^ antigen by patient-derived melanoma cells. Of the five different patient-derived melanoma cell lines tested for gp100^mel^ protein and HLA-A*03:01 expression only Mel63a cells proved to be HLA-A*03:01^+^/gp100^mel +^ (Fig. 3A,B). Immunoblotting revealed that Mel63a cells mostly expressed intermediate-type proteasomes, expressing all standard and IFN-γ inducible proteasome subunits except the β2i subunit (Fig. 3B). Interestingly, the Mel63a cells elicited a strong IFN-γ release by the gp100^mel^_47–52/40–42_ specific T-cell clone, suggesting that the spliced peptide gp100^mel^_47–52/40–42_ can indeed be produced from the full-length gp100^mel^ protein (Fig. 3C). This proved that the spliced gp100^mel^_47–52/40–42_ peptide identified by applying SpliceMet in *in vitro* experiments represented a natural tumor epitope produced from the full-length gp100^mel^ membrane protein and was presented on the surface of gp100^mel^/HLA-A*03:01^+^ patient-derived melanoma cells.

### The gp100^mel^_47–52/40–42_ epitope is generated by the ERAD pathway

**Figure 4:**
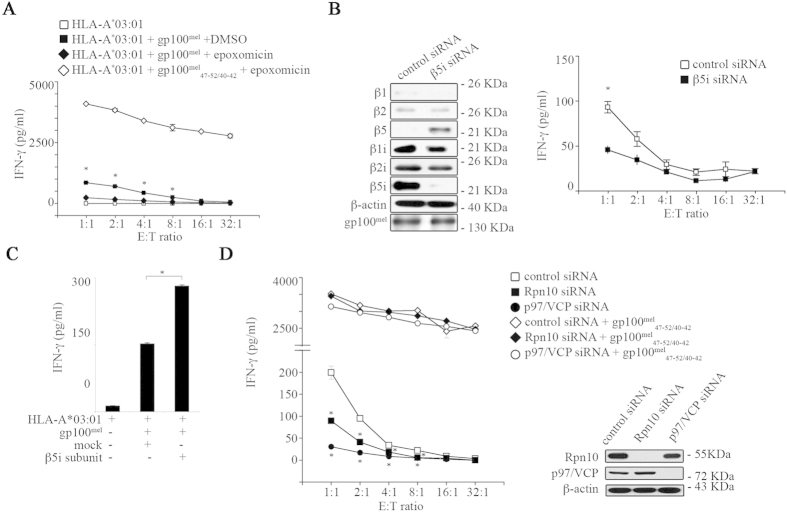
Ub-proteasome- and ERAD-dependent gp100^mel^_47–52/40–42_ epitope production is enhanced by β5i subunit. (**A**) HeLa cells were transfected with HLA-A*03:01 and gp100^mel^, and were subjected to epoxomicin treatment (250 nM; 2 hrs) or DMSO (control) and compared to HeLa cells transfected with HLA-A*03:01 treated with epoxomicin (250 nM; 2 hrs) and pulsed with the synthetic peptide gp100^mel^_47–52/40–42_ (50 μM). Values are means and SD of 2 replicates in a representative assay of 2 independent experiments. Significant differences (between the samples with or without epoxomicin) in the means of replicates are marked by * (student t-test; E:T ratio: 1:1, p = 0.002; 2:1, p = 0.001; 4:1, p < 0.001; 8:1, p < 0.001). (**B**) HeLa 33/2 cells were exposed to β5i subunit or control siRNAs (30 nM for 24 hrs) and subsequently transfected with gp100^mel^. Cell extracts were analyzed by western blotting (representative of 4 independent experiments). β5i subunit-depleted HeLa 33/2 cells were subjected to a 16 hr CTL assay. Values are means and SD of 2 replicates in a representative assay of 4 independent experiments. Significant differences in the means of replicates are marked by * (student t-test; p = 0.047). (**C**) HeLa cells were transfected with plasmids encoding HLA-A*03:01, gp100^mel^ full-length protein, β5i subunit or the empty vector. The HeLa cell ability to process the gp100^mel^_47–52/40–42_ was assessed by CTL assay. Values are means and SD of 2 replicates in a representative assay of 4 independent experiments. Significant difference in the means of replicates is marked by * (student t-test; p = 0.001). (**D**) HeLa cells were exposed to 30 nM siRNA specific for the 19S Rpn10 subunit or p97/VCP (or control siRNA) prior to transfection with the HLA-A*03:01 and the full-length gp100^mel^ protein plasmids and pulsed or not pulsed with the synthetic peptide gp100^mel^_47–52/40–42_ (30 μM). Values are means and SD of 2 replicates in a representative assay of 3 independent experiments. Significant differences in the means of replicates are marked by * (student t-test) for Rpn10 siRNA (E:T ratio: 1:1, p = 0.038; 2:1, p = 0.029; 4:1, p = 0.047; 8:1, p = 0.002) and for p97/VCP siRNA (E:T ratio: 1:1, p = 0.016; 2:1, p = 0.020; 4:1, p = 0.024; 8:1, p = 0.001). The knock-down efficiencies were monitored by determining the steady-state level of Rpn10 or p97/VCP proteins by western blotting.

To exclude the possibility that generation of the gp100^mel^_47–52/40–42_ peptide was restricted to a specific cell type and to study the contribution of individual proteasome subunits in the production of the peptide, HeLa cells were transfected with plasmids encoding the HLA-A*03:01 protein and a full-length gp100^mel^-*myc*/His fusion protein. As shown in Fig. 4A, HeLa cells efficiently presented the gp100^mel^_47–52/40–42_ epitope in a proteasome-dependent manner providing evidence that epitope generation was not dependent on a specific cell type. The *in vitro* experiments had indicated a driving role for the two different β5 subunits in the generation of the gp100^mel^_47–52/40–42_ epitope (see Fig. 1F,G). To study the role of the β5-subunit isoforms in a cellular context, we monitored gp100^mel^_47–52/40–42_ epitope presentation in HeLa 33/2 cells, which constitutively express i-proteasomes, using a siRNA approach. The siRNA-induced silencing of β5i subunit expression resulted in its substitution by the standard β5 subunit within the 20S proteasome complex (Fig. 4B). This subunit substitution led to a remarkable reduction of gp100^mel^_47–52/40–42_ presentation underlining the importance of the β5i subunit for this splicing reaction (Fig. 4B). Corroborating this result, over-expression of the β5i subunit in HeLa cells and concomitant formation of β5i/ β1/β2 subunits containing intermediate type 20S proteasomes led to a strong increase in gp100^mel^_47–52/40–42_ presentation (Fig. 4C). Interestingly, the proteasome subunit composition found in Hela cells overexpressing β5i subunit was similar to that observed in Mel63a cells suggesting that the intermediate type proteasomes carrying β5i, β2 and β1 subunits are best equipped for the generation and presentation of this spliced epitope.

Because gp100^mel^ is a membrane protein we also asked whether generation of the gp100^mel^_47–52/40–42_ epitope from gp100^mel^ protein resulted from the ER-associated ubiquitin-dependent protein degradation pathway (ERAD). To test this, expression of the ubiquitin-receptor subunit Rpn10 residing in the 19S complex and of the ERAD component p97/VCP were silenced by siRNA. As shown in (Fig. 4D) silencing of either Rpn10 or p97/VCP expression significantly impaired the endogenous gp100^mel^_47–52/40–42_ epitope presentation providing evidence that PCPS takes place as part of normal ubiquitin-dependent protein degradation and can also involve the ERAD pathway.

### PBMCs of melanoma patients harbor spliced epitope-specific T cells

Because the gp100^mel^_47–52/40–42_ epitope had been identified using an open unbiased algorithm-aided reverse immunology approach, the possibility still remained that its identification and generation may have been the result of the experimental set ups. Hence, it was important to validate whether the gp100^mel^_47–52/40–42_ peptide was also generated *in vivo* and that melanoma patients possessed CD8^+^ T cells directed against this epitope.

**Figure 5:**
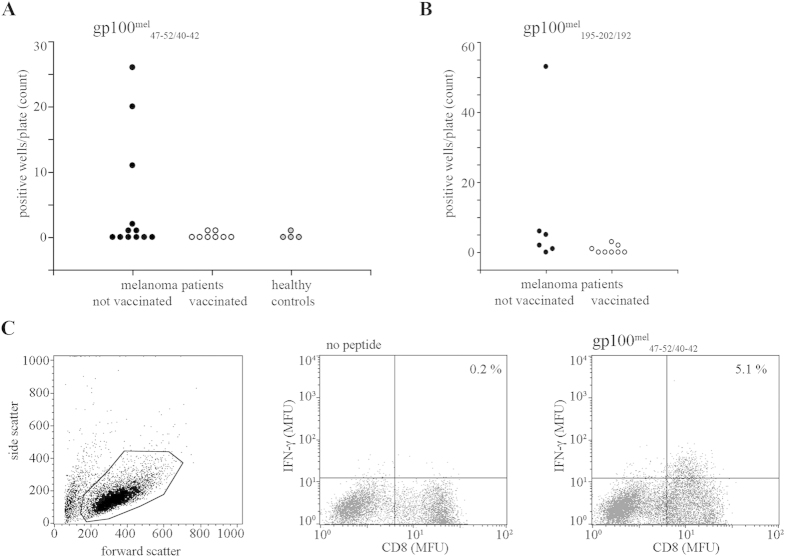
IFN-γ responses to the gp100^mel^_47–52/40–42_ and gp100^mel^_195–202/192_ spliced peptides in HLA-A*03:01^+^ healthy controls and melanoma patients. PBMCs from healthy controls and patients with melanoma vaccinated or not with dendritic cells pulsed with the gp100^mel^_209–217_ peptide were cultured in 96-well plates and stimulated with 30 μM gp100^mel^_47–52/40–42_ (**A**) or gp100^mel^_195–202/192_ (**B**) synthetic peptides. After 10 days of culture, the presence of CTLs specific for gp100^mel^_47–52/40–42_ and gp100^mel^_195–202/192_ epitopes in each well was assessed by re-stimulating the cells with the gp100^mel^_47–52/40–42_ or gp100^mel^_195–202/192_ peptide in a 16 hr assay in the presence of CD28/CD49d co-stimulatory molecules. A negative control without peptide was included in all experiments. After 16 hrs, supernatants were collected and tested for their IFN-γ content by ELISA. Shown is the number of wells per plate in which the IFN-γ secretion upon peptide stimulation was larger than 100 pg/ml and at least twice as large as that detected without peptide for each patient and/or control. (**C**) Validation of the ELISA outcome on the representative healthy control well showing the larger IFN-γ response by CD8^+^ T cells in presence of the target spliced epitope. CTLs were double stained for CD8 and intracellular IFN-γ by FACS analysis. The side/forward scatters chart is reported in the left panel. Cells included in the analysis are marked with a circle and are shown in the two right panels, where CTLs were co-cultivated for 16 hrs with antigen presenting cells pulsed or not pulsed with the 30 μM gp100^mel^_47–52/40–42_ synthetic peptide prior the FACS analysis.

For proof of principal, we therefore tested whether HLA-A*03:01^+^ melanoma patients possessed CD8^+^ T cells directed against the gp100^mel^_47–52/40–42_ peptide (Fig. 5A) and/or the HLA-A*03:01-restricted spliced peptide gp100^mel^_195–202/192_ [RSYVPLAH][R] (Fig. 5B), which was previously shown to trigger a T cell response in melanoma patients^18^. In total, PBMCs from 20 randomly chosen melanoma patients and 4 healthy controls were analyzed. Interestingly, 6/12 untreated patients and 2/8 vaccinated patients displayed T cells reactive against the gp100^mel^_47–52/40–42_ epitope (Fig. 5A). Only 1 out of 4 healthy controls had a measurable specific response toward the same spliced epitope (Fig. 5A), as further confirmed by FACS analysis (Fig. 5C). On the other hand, 5/6 untreated and 2/8 vaccinated melanoma patients contained T cells recognizing the gp100^mel^_195–202/192_ peptide (Fig. 5B). This data demonstrated that our algorithm-based reverse immunology approach is a potent strategy to identify new spliced epitopes. They also suggested that proteasome-catalyzed generation of spliced antigenic peptides is not an isolated singular event but is part of the physiological proteasome-dependent epitope generation process, which elicits specific CD8^+^ T cell responses *in vivo* in melanoma patients.

### Spliced and non-spliced epitopes are presented at the cell surface in comparable amount

Despite being an important issue in judging the immune relevance of spliced epitopes, there exists no study comparing the relative presentation efficacy of different spliced epitopes with each other or with non-spliced epitopes. The gp100^mel^ antigen seemed be ideally suited for such an analysis because the spliced peptides gp100^mel^_47–52/40–42_ (HLA-A*03:01), gp100^mel^_40–42/47–52_ (HLA-A*32:01), gp100^mel^_195–202/192_ (HLA-A*03:01) and the non-spliced gp100^mel^_209–217_ (HLA-A*02:01) were all generated from the same protein. To extend the analysis to a second and gp100^mel^-independent non-spliced epitope we also included the tumor-associated HLA-A*02:01-restricted epitope NY-ESO-1_157–165_^26^ into the comparison. However, to perform such comparison, different HLA-A peptide binding affinities, HLA expression levels as well as the different affinities of the CD8^+^ T cells have to be taken into consideration.

**Figure 6:**
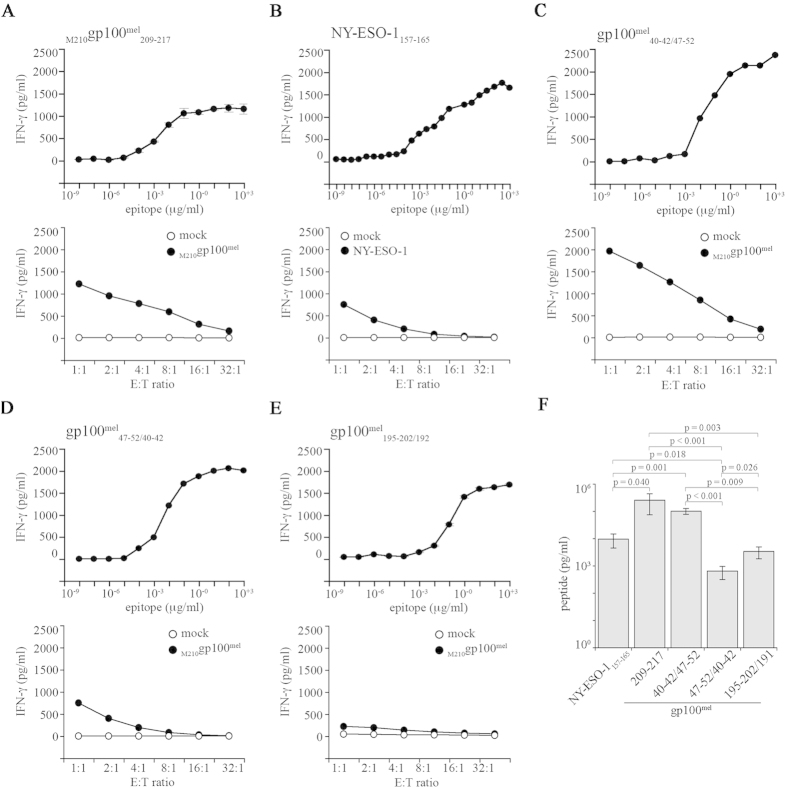
The processing efficacy of the gp100^mel^–derived spliced epitopes is comparable to that of the non-spliced _M210_gp100^mel^_209–217_ and NY-ESO-1_157–165_ epitopes. (**A–E**) The IFN-γ-release by specific CD8^+^ T cell clones exposed to HLA-A*03:01/-A*02:01/-A*32:01^+^ HeLa cells (E:T = 1:1) pulsed with different concentrations of the synthetic peptides (upper panels) or HLA-A*03:01/-A*02:01/-A*32:01^+^ HeLa cells transfected with the target antigen (lower panels) is shown for the epitopes _M210_gp100^mel^_209–217_ (**A**), NY-ESO-1_157–165_ (**B**), gp100^mel^_40–42/47–52_ (**C**), gp100^mel^_47–52/40–42_ (**D**) or gp100^mel^_195–202/192_ (**E**). The data are the mean and bars the SD of three replicates of a representative assay of independent experiments (n = 2–6). (**F**) We here report a marker of the amount of epitope presented on the cell surface of HLA-A*03:01/-A*02:01/-A*32:01^+^ HeLa cells transfected with the target antigen. By titrations (upper panels in **A–E**) we calculated the amount of synthetic peptide pulsed on to HeLa cells, which triggered the release of IFN-γ amount corresponding to the IFN-γ released by the specific CD8^+^ T cell clones exposed to HLA-A*03:01/-A*02:01/-A*32:01^+^ HeLa cells transfected with the target antigen and cultivated in an E:T ratio = 4:1 (see lower panels in **A–E**). Values are the means and bars the SEM of independent experiments (n = 2–7; each experiment with three independent replicates). Significant differences in the means are marked and the corresponding by p values are reported (we applied the Mann-Whitney test with Bonferroni correction for multiple comparison). The IFN-γ-release in the medium was measured by ELISA after 16 hrs co-culture.

Therefore we first performed titration experiments with HeLa cells expressing the target HLA-A molecules, pulsed with the antigenic peptides and co-cultivated them with the respective peptide-specific CD8^+^ T-cell clones (Fig. 6A–E upper panels). By measuring the resulting IFN-γ release, informative titration curves where obtained for all antigenic peptides (Fig. 6B–E) but the non-spliced gp100^mel^_209–217_ epitope (data not shown). Because gp100^mel^_209–217_ is known be a poor HLA-A*02:01 binder, we consequently performed the titration experiments with the previously described _M210_ gp100^mel^_209–217_ peptide, in which the anchor residue T_210_ of gp100^mel^ was exchanged with a Methionine for better HLA-A *02:01 binding. T cell assays were performed with transduced CD8^+^ T cells expressing the corresponding TCR. In this case satisfactory titration curves were obtained (Fig. 6A). We next transfected HeLa^HLA-A*03:01+^ cells with plasmids encoding the _M210_gp100^mel^ variant, or the NY-ESO-1 proteins together with the three HLA-A*32:01, -A*02:01 and -A*03:01molecules. The transfected cells were then tested for their ability to activate CD8^+^ T-cells specific for each of the peptides as outlined above (Fig. 6A–E lower panels).

We considered the measured IFN-γ release obtained with an 4:1 E:T ratio and determined the corresponding peptide concentrations on the basis of the titration experiments. We chose the 4:1 E:T ratio to obtain IFN-γ release values in the log-phase of the titration curves. By applying this method we obtained a semi-quantitative comparison of the amount of non-spliced *vs* spliced epitopes generated by proteasomes of HeLa cells and presented by MHC class I molecules and. As shown in Fig. 6F the amounts of the spliced gp100^mel^_40–42/47–52_ epitope and of the non-spliced epitope _M210_gp100^mel^_209–217_ were comparable and the amounts of the spliced gp100^mel^_40–42/47–52_ epitope exceeded those of all other epitopes analyzed. In contrast, cell surface expression of the new spliced gp100^mel^_47–52/40–42_ epitope was significantly lower, which is in agreement with our observation that s-proteasomes produce only relatively low amounts of the gp100^mel^_47–52/40–42_ epitope. Interestingly, also the expression of the non-spliced NY-ESO-1_157–165_ epitope was lower than that of the gp100^mel^_40–42/47–52_ and _T210M_gp100^mel^_209–217_ epitopes and comparable with the spliced gp100^mel^_195–202/192_ epitope, requiring C-terminal trimming. As a control, we compared the CD8^+^ T-cell response towards gp100^mel^_40–42/47–52_ and gp100^mel^_47–52/40–42_ epitopes in HeLa cells expressing the wild-type gp100^mel^ and the mutated _T210M_gp100^mel^ proteins in two independent experiments. No difference emerged confirming that the use of the _T210M_gp100^mel^ variant did not affect the generation of the distant spliced epitopes (Fig. S5).

In summary, these experiments demonstrate that generation and presentation of spliced epitopes occurs with efficacies similar to what is observed for canonical non-spliced epitopes and do not represent exceptions with respect to their immune relevance.

## Discussion

Generation of spliced epitopes is an additional proteasome-dependent activity, contributing to the pool of MHC-class I presented antigenic peptides. A number of features of the proteasome-dependent splicing events indicate that peptide splicing is not an accidental side reaction of proteasome activity but rather an authentic post-translational modification. One important aspect of PCPS is the fidelity of the process because to trigger a CD8^+^ T cell response a spliced peptide must be repeatedly generated from a given substrate protein. Thus, for a functional purpose of the resulting splicing products PCPS must be a finely tuned event^27^. However, a proteasome-catalyzed splicing event is difficult to detect because it requires the complementary information on gene sequence and processing events. Because of that, spliced epitopes have so far escaped wide spread identification using available databases in combination with mass spectrometric analyses. We here applied an algorithm aided reverse immunology approach, which *a priori* does not rely on available patient-derived CD8^+^ T cells and therefore greatly facilitates the identification of new spliced epitopes.

As part of our analyses the spliced gp100^mel^_47–52/40–42_ peptide was identified as a new HLA-A*03:01-restricted tumor epitope expressed on patient-derived gp100^mel+^ melanoma cells. Interestingly, the gp100^mel^_47–52/40–42_ peptide was generated *in vitro* from the same gp100^mel^_40–52_ 13-mer polypeptide substrate as the previously reported spliced gp100^mel^_40–42/47–52_ epitope^12^ but with the same peptide fragments being arranged in reverse order. This disclosed that in addition to the well-established transpeptidation reaction^11^,^12^,^14^ PCPS could also involve condensation reactions that *in vitro* are independent of prior proteasomal peptide-bond hydrolysis. Production of the spliced antigenic peptide in a condensation reaction could also be observed *in cellulo* when the 13-mer gp100^mel^_40–52_ polypeptide substrate was expressed in HeLa cells as part of an ubiquitin fusion protein. Condensation reactions performed by proteinases have only been reported to occur *in vitro* under defined diffusion-limiting conditions. The gated barrel-shaped catalytic cavity of the 20S proteasome hindering rapid diffusion of the generated peptide as well as the catalytic activity of Thr1 via the formation of an ester-bond intermediate may however explain why proteasomes are able to perform condensation reactions also in a cellular environment with relative efficacy. In fact, in case the peptide ligands are held in the right orientation in close proximity, this does not even require catalysis. It is however not possible to determine the extent to which condensations reactions participate *in cellulo* to the overall production of the gp100^mel^_47–52/40–42_ peptide from the full-length gp100^mel^ protein.

Theoretically, only relatively few MHC-class I-peptide complexes have to be exposed at the cell surface to trigger an immune response^28^ and T cells directed against spliced epitopes have been identified among a subset of tumor infiltrating lymphocytes^10^. Nevertheless, the notion that spliced epitopes represented a curiosity still prevailed and doubts remained whether PCPS was a sufficiently frequent event to play any significant role in MHC class I-restricted immune responses. Importantly, PCPS does not randomly produce an unlimited number of all theoretically possible spliced peptides^14^,^23^. Rather, PCPS seemed to be restricted by certain sequence characteristics of the substrate protein, guaranteeing a high reproducibility in the generation of spliced epitopes^14^. But similar to the well-characterized proteasome-generated non-spliced epitopes for which reliable rules are still missing, general sequence rules governing spliced epitope generation are so far unresolved.

An important issue with respect to their immune relevance and a still unresolved question was whether spliced epitope presentation is at all comparable with that of non-spliced conventional tumor epitopes. Therefore, in a semi-quantitative analysis we also compared for the first time the relative efficacy of endogenous presentation of three different gp100^mel^ derived spliced epitopes with that of two non-spliced tumor epitopes. Relative cell surface presentation efficacy of the three gp100^mel^ derived spliced epitopes analyzed here differed considerably among each other. In particular, cell surface expression of the spliced gp100^mel^_40–42/47–52_ epitope was significantly superior to the presentation of the newly identified gp100^mel^_47–52/40–42_ epitope, which is composed of the same splice-reactants but in reverse order. This difference may be explained in that the reverse order peptide-ligation by either transpeptidation or condensation is less efficient and/or that the generation of the gp100^mel^_47–52/40–42_ epitope is not favored by s-proteasomes. Importantly, our data show for the first time that the overall relative cell surface expression levels of the spliced epitopes is comparable to that of the non-spliced gp100^mel^_209–217_ and NY-ESO-1_157–165_ epitopes. This suggests that with regard to their relative presentation efficacy proteasome-generated spliced epitopes exert features almost identical to those known for non-spliced epitopes and do not greatly differ from what is known for canonical epitopes.

Considering that the spliced gp100^mel^_47–52/40–42_ epitope was identified in an unbiased reverse immunology approach and the fact that the PBMCs from approximately 50% of randomly chosen untreated melanoma patients contained CD8^+^ T cells that were stimulated by the gp100^mel^_47–52/40–42_ peptide suggested that generation of spliced epitopes is indeed a rather frequent event. Corroborating this result, five out of six untreated patient samples turned out to contain memory CD8^+^ T cells responsive to the spliced HLA-A*03:01-restricted epitope gp100^mel^_195–202/191_ [RSYVPLAH][R] that had initially been identified through the analysis of tumor infiltrating lymphocytes^18^. That a fewer number of samples obtained from melanoma patients having undergone vaccination with dendritic cells were responsive to the spliced antigenic peptides might be owed to the fact that they contained a relatively larger percentage of T cells directed against the non-spliced peptides, which were used for vaccination. Although our assay conditions are not designed to prime naïve T-cells in that dendritic cells were in low number and not activated, we can not completely exclude that the immune response towards the spliced epitopes may in part be due to priming and the activation of naïve CD8^+^ T cells during the *in vitro* assays

Supporting the conclusion that generation of MHC-class I ligands by PCPS was not a rare event, we also identified, by applying our SpliceMet-supported reverse immunology approach, a significant number of potential spliced epitopes derived from human gp100^mel^ or *Listeria monocytogenes* LLO antigens and with high HLA-A and/or -B binding affinity, which warrant further immunological characterization (Table 1).

Our data also demonstrated that PCPS is intrinsically linked to ubiquitin-dependent proteasomal protein turnover and proteasomal hydrolysis events and that it was not a rare side reaction. Since peptide exhibiting features suitable for binding to MHC class I molecules seem to occur in among spliced peptides with a certain preference^14^, the relative probabilities for generation of a spliced epitope or a non-spliced epitope from a given substrate are most likely not very different. Therefore, the pool of spliced antigenic peptides available for selection by the antigen presentation machinery may be significantly larger than previously estimated. In fact, as shown here for the gp100^mel^ protein PCPS also seems to enlarge the HLA haplotype-restriction of antigenic peptides that can be produced from a single substrate protein thereby broadening the cellular immune response.

## Materials and Methods

A detailed Material and Method section is disclosed in the Supplementary information.

### Peptide synthesis and quantification

The sequence enumeration for the polypeptides gp100_40–52_ [RTKAWNRQLYPEW], gp100^mel^_209–217_ [IMDQVPFSV], gp100^mel^_195–202/92_ [RSYVPLAH][R] are referred to the human protein gp100^PMEL17^, for the peptide [SLLMWITQC] to the NY-ESO-1 human protein. All peptides were synthesized as described elsewhere^23^. LC-MS analyses were performed as previously described^14^. Database searching was performed using the SpliceMet’s ProteaJ algorithm^23^. Quantification of proteasome-generated non-spliced and spliced peptides (Fig. 1, Fig. S2) was carried out by applying the QME method to LC-MS analyses as described elsewhere^14^. For semi-quantitative studies of the amount of the two spliced peptides [QLYPEW][RTK] and [RTK][QLYPEW] (Fig. 1F,G), the heavy peptides QLYPE^+6^WRTK and RTK^+6^QLYPEW were used as internal standards. The limit of quantification was set to 10 fmol upon preliminary experiments with synthetic peptide titration

### *In vitro* processing of synthetic peptides

The synthetic peptide gp100^mel^_40–52_ (40 μM) was digested by 3 μg 20S proteasomes in 100 μl TEAD buffer over time at 37 °C. In the *in vitro* hydrolysis-independent proteasome-mediated experiments (Fig. 1F,G) 5 μg 20S proteasomes were incubated in 100 μl TEAD buffer in presence of 100 μM peptides for 20 hours. For the experiments with the inhibitor PR-893, PR-924 (Onyx Pharmaceuticals, US), YU-102 and LU-102^25^ the specific binding to the proteasome subunits was monitored.

### 20S proteasome purification

20S proteasomes were purified from LCLs and T2 cell line as previously described^29^. 20S proteasome purified from human erythrocytes and spleen were purchased from BioMol.

### Cell culture

Cells used in this study include LCLs, T2 cells, HeLa cells, HeLa 33/2 cells which were all previously described^9^,^30^,^31^. The melanoma cell lines Ma-Mel15a, Ma-Mel18, Ma-Mel21a and Ma-Mel63a were established from tumor metastasis and grown in RPMI 1640 with 10% FCS, 1% L-glutamine and 1% penicillin. The CTL clones K631 1C specific for the HLA-A*03:01-restricted gp100^mel^_47–52/40–42_ epitope and RG39 specific for the HLA*02:01-restricted NY-ESO-1_157–165_ epitope were prepared according to the procedure of Fonteneau *et al*.^32^. The LG2-37.7.14 and M45-3B CD8^+^ T cell clones specific for the HLA-A*32:01-restricted gp100^mel^_40–42/47–52_ and HLA-A*03:01-restricted gp100^mel^_195–202/192_ spliced peptides, respectively were described elsewhere^12^,^18^. The gp100^mel^-specific TCR-transduced T cells were generated as previously described^33^,^34^.

### HLA-A03:01-peptide binding affinity

Binding affinity of synthetic peptides were computed with different peptide concentration in a standard binding affinity assay measured by flow cytometry as described elsewhere^29^, using HeLa or T2 cells transfected with the HLA-A*03:01-expressing plasmid.

### Plasmid and siRNA transfection

Expression vectors used in thy study include the pcDNA3.1 (HLA-A*02:01, HLA-A*32, β5i), pcDNA3.1/myc-HIS (gp100^mel^, NY-ESO-1) and the pcDNA3.1(+)/Zeo (HLA-A*03:01, HA-Ub/gp100^mel^_40–52_) plasmids. Transfection of HeLa and 33/2 cells or T2 cells was performed using Lipofectamin2000 or Amaxa-Nucleofactor technology, respectively, according to the manufacturer’s instructions. All siRNA (control, Rpn10 and p97/VCP) used in this study were purchased from Dharmacon and introduced into HeLa or 33/2 cells as previously described^31^.

### Antibodies and western blotting

Cell extracts were prepared as previously described^31^. Proteins were resolved by SDS-PAGE and blotted with β1i, β2i, β5i, β1, β2, β5, α6 proteasome subunits, Rpn10, HA, gp100^mel^, p97/VCP and β-actin (as a loading control).

### CTL assays and screening of gp100_47–52/40–42_-specific CD8^+^ T cells in HLA-A*03:01 healthy donors’ and melanoma patients’ PBMCs

Targets cells were serially diluted and co-cultured with a fixed amount of T cells, resulting in graded effector-to-target (E:T) ratio for 12 h prior to the measurement of IFN-γ release by ELISA. PBMCs from healthy donor or patients with melanoma were pulsed with 30 μM of the gp100^mel^_47–52/40–42_ synthetic peptide in RPMI medium supplemented with 8% human AB serum, 1% penicillin and IL-2 (50 U/ml). After 10 days of culture, their capacity to produce IFN-γ in response to the cognate peptide was measured by ELISA.

## Statistical Analysis

Data were tested for normality distribution and homoscedasticity by Kolmogorov-Smirnov, Shapiro-Wilk and Levene tests. To identify significant difference between groups reported in Fig. 6, Kruskal-Wallis followed by Mann-Whitney tests with Bonferroni Post Hoc correction for multiple comparisons were applied. Otherwise, paired and unpaired Student t-tests were carried out. Descriptive statistics were carried out with SPSS (version 17) and R; a *p*-value <= 0.05 was considered statistically significant.

## Additional Information

**How to cite this article**: Ebstein, F. *et al*. Proteasomes generate spliced epitopes by two different mechanisms and as efficiently as non-spliced epitopes. *Sci. Rep*. **6**, 24032; doi: 10.1038/srep24032 (2016).

## Supplementary Material

Supplementary Information

## Figures and Tables

**Table 1:** Potential proteasome-catalyzed spliced antigenic peptides derived from human gp100^mel^_35–57_, gp100^mel^_201–230_ and LLO_291–317_ substrates and identified by a reverse immunology approach.

Spliced peptide	Sequence	HLA-A and/or -B (IC50, nM)
***substrate*****gp100**^**mel**^_**35–57**_***–*****VSRQLRTKAWNRQLYPEWTEAQR**		
gp100^mel^_35–39/35–39_	[VSRQL] [VSRQL]	B*27:05 (148)
gp100^mel^_45–52/35–37_	[NRQLYPEW][VSR]	A*31:01 (38)
gp100^mel^_47–52/40–42_	[QLYPEW][RTK]	A*03 (86)
gp100^mel^_49–52/35–39_	[YPEW][VSRQL]	B*07:02 (18)
***substrate*****gp100**^**mel**^_**201–230**_***–*****AHSSSAFTITDQVPFSVSVSQLRALDGGNK**		
gp100^mel^_201–207/201–207_	[AHSSSAF][AHSSSAF]	A*24:03 (11), B*15:01 (120), B*15:03 (113)
gp100^mel^_210–218/220–222_	[TDQVPFSVS][SQL]	B*07:02 (94), B35:01 (182), B39:01 (133)
***substrate*****LLO**_**291–317**_ ***–*****AYISSVAYGRQVYLKLSTNSHSTKVKA**		
LLO_291–293/295–302_	[AYI][SVAYGRQV]	B*15:17 (31)
LLO_291–294/297–304_	[AYIS][AYGRQVYL]	A*02:02 (199), A*02:11 (89), A*02:50 (115), B*15:17 (50).
LLO_291–298/291–292_	[AYISSVAY][AY]	A*01:01 (121), A*29:02 (8), A*30:02 (20), A*68:01 (68), A*80:01 (38), B*15:01 (66), B*15:03 (100), B*35:01 (22)
LLO_291–298/291–293_	[AYISSVAY][AYI]	A*30:01 (145), B*15:17 (52), B*58:01 (25)
LLO_299–304/291–293_	[GRQVYL][AYI]	B*27:05 (99)

Proteasome-generated spliced epitopes were identified by applying SpliceMet^23^ on the proteasome-mediated digestion of the synthetic substrates gp100^mel^_35–57_, gp100^mel^_201–230_ and *Listeria monocytogenes* LLO_291–317_ as previously reported^14^. Afterwards, the spliced antigenic peptide list was analyzed for binding affinity to human MHC variants using the web-available ANN algorithm^24^. In agreement with our experience, only spliced epitopes with IC50 < 200 nM were considered as potential epitopes. We included in the list the N-terminal-extended precursors of the potential minimal epitopes. For each potential proteasome-catalyzed spliced epitope the human HLA variant and the predicted IC50 (nM) are disclosed.
