# Supplementary material for: Proteasomes generate spliced epitopes by two different mechanisms and as efficiently as non-spliced epitopes
Source: Sci Rep. 2016 Apr 6;6:24032. doi: 10.1038/srep24032 (PMC4822137; doi:10.1038/srep24032)
Supplement: Supplementary Information [file srep24032-s1.pdf]

Proteasomes generate spliced epitopes by two different mechanisms and as efficiently as non-spliced epitopes

Ebstein F, Textoris-Taube K, Keller C, Golnik R, Vigneron N, Van den Eynde BJ, Schuler-Thurner B, Schadendorf D, Lorenz FKM, Uckert W, Urban S, Lehmann A, Albrecht-Koepke N, Janek K, Henklein P, Niewienda A, Kloetzel PM, Mishto, M.

Supplementary information

Fig. S1	MS identification of proteasome-catalyzed spliced peptides $gp100^{mel}_{40-42/47-52}$, $gp100^{mel}_{47-52/40-42}$, $gp100^{mel}_{47-52/40-46}$ in *in vitro* digestion
Fig. S2	Transpeptidation and condensation efficiency differ from s- to i-proteasome
Fig. S3	MS identification of the spliced peptide $gp100^{mel}_{47-52/40-42}$ in hydrolysis-independent reaction
Fig. S4	Generation and characterisation of the $CD8^{+}$ T cell clone (K631 1C) specific for the $gp100^{mel}_{47-52/40-42}$ spliced epitope
Fig. S5	T210M $gp100^{mel}$ substitution does not affect the *in cellulo* presentation of the spliced epitopes $gp100^{mel}_{40-42/47-52}$, $gp100^{mel}_{47-52/40-42}$.

Detailed Materials and Methods

Peptide synthesis and quantification. The sequence enumeration for the polypeptides $gp100_{40\text{-}52}$ [RTKAWNRQLYPEW], $gp100^{mel}_{209\text{-}217}$ [IMDQVPFSV], $gp100^{mel}_{195\text{-}202/92}$ [RSYVPLAH][R] are referred to the human protein $gp100^{PMEL17}$, for the peptide [SLLMWITQC] to the NY-ESO-1 human protein. All peptides were synthesized using Fmoc solid phase chemistry and the purity of synthetic peptides was tested by amino acid analysis as described [1]. For the heavy peptides (**Fig. 1*E-G***) the following heavy amino acids from Euriso-Top GmbH were used: L-Glutamic acid-N-Fmoc, Gamma-Tert-Butyl-Ester (U-13C5/15N) and L-Lysine-alpha-N-Fmoc, Epsilon-n-t-Boc (U-13C6). LC-MS analyses were performed as described [2] with the ESI-ion trap instrument DECA XP MAX (ThermoFisher Scientific, USA), the LTQ Orbitrap XL MS (Thermo) coupled with nanoUPLC Aquity (Waters) and the MALDI-TOF/TOF mass spectrometer 4700 Proteomics Analyzer (Applied Biosystems, Framingham, MA, USA) offline coupled with a Thermo Scientific Dionex UltiMate 3000 RSLC system and Probot fractionation device (ThermoScientific, Idstein, Germany). Database searching was performed using the SpliceMet's ProteaJ algorithm [1]. Quantification of proteasome-generated non-spliced and spliced peptides (**Fig. 1, Fig. S2**) was carried out by applying the QME method to LC-MS analyses as described elsewhere [2]. For semi-quantitative studies of the amount of the two spliced peptides [QLYPEW][RTK] and [RTK][QLYPEW] (**Fig. 1*F, G***), the heavy peptides $QLYPE^{+6}WRTK$ and $RTK^{+6}QLYPEW$ were used as internal standards. Identity of the proteasome-generated spliced peptides was verified by MS/MS analysis and by comparison of the retention time with the internal standard peptides. The quantification was performed as previous described [2]. The total sum of the isotopic peak areas at *m/z* 1220.7, 1221.7, 1222.7 of 2-3 MS spectra was compared with those of the heavy peptides and their relative ratio was calculated. The estimated amount of the spliced peptides corresponded to the injected sample volume (9 µl reaction sample + 1 µl internal standard) on the column. The limit of quantification was set to 10 fmol upon preliminary experiments with synthetic peptide titration (data not shown). This correlated to a mono-isotopic peak with a signal to noise ratio of at least 150.

In vitro* processing of synthetic peptides**. The synthetic peptide $gp100^{mel}_{40\text{-}52}$ (40 µM) was digested by 3 µg 20S proteasomes in 100 µl TEAD buffer (Tris 20 mM, EDTA 1 mM, NaN_3 1 mM, DTT 1 mM, pH 7.2) over time (0 – 4 hrs) at 37 °C. All experiments were repeated and measured at least twice. In the *in vitro* hydrolysis-independent proteasome-mediated experiments (**Fig. 1*F, G) 5 µg 20S proteasomes were incubated in 100 µl TEAD buffer in presence of 100 µM peptides for 20 hours. For the experiments with the inhibitor PR-893, PR-924 (Onyx Pharmaceuticals, US), YU-102 and LU-102 [3] the specific binding to the proteasome subunits was monitored (data not shown).

20S proteasome purification. 20S proteasomes were purified from LCLs and T2 cell line as previously described [4]. 20S proteasome purified from human erythrocytes and spleen were purchased from BioMol.

Cell cultures. Cells were checked for mycoplasma contamination. LCLs are human B lymphocytes immortalized with Epstein Barr virus. T2 cell line is a human T cell leukemia/B cell line hybrid defective in TAP1/TAP2 (transporter associated with antigen presentation) and β1i/β5i subunits. T2 cell line and erythrocytes contain only s-proteasomes, whereas spleen and LCLs posses proteasomes carrying mainly immuno-subunits [5,6]. HeLa cells were cultivated in Iscove medium supplemented with 10% FCS, 1% L-glutamine and 1% penicillin (Biochrom). The HeLa 33/2 cell line constitutively expresses the three β5i, β1i and β2i immuno-subunits. It was maintained in Iscove medium supplemented with 10% FCS, 1% L-glutamine, 1% of a combination of penicillin and streptomycin as well as 2 µg/ml puromycin and 300 µg/ml hygromycin (PAA Laboratories). The melanoma cell lines Ma-Mel15a, Ma-Mel18, Ma-Mel21a and Ma-Mel63a were established from tumor metastasis after informed consent of the patient. Melanoma cell lines, LCL and T2 cell lines are grown in RPMI 1640 (Biochrom) with 10% FCS, 1% L-glutamine and 1% penicillin. The CTL clone K631 1C specific for the HLA-A*03:01-restricted $gp100^{mel}_{47-52/40-42}$ epitope was prepared according to the procedure of Fonteneau *et al.* [7]. In brief, $CD8^+$ T cells from a HLA-A*03:01$^+$ healthy donor were stimulated with dendritic cells preloaded with 50 µM $gp100^{mel}_{47-52/40-42}$ synthetic peptide. After two rounds of stimulation in the presence of IL-6 (10 ng/ml), IL-12 (10 ng/ml) and IL-2 (50 U/ml) (Miltenyi Biotec), the primed $CD8^+$ T cells were screened in a standardized 6 hrs WEHI-13 based-assay for their capacity to secrete TNF-α upon re-stimulation with the cognate epitope. In this assay TNF-α release was measured by lysis of WEHI-13 cells. The frequency of the specific $CD8^+$ T cells present in the responsive micro-cultures was determined by intracellular double staining for CD8 and IFN-γ using flow cytometry. The micro-culture well #136, which contained ~10% of $CD8^+$ T cells specific for $gp100^{mel}_{47-52/40-42}$ (**Fig. S4**) was used for the generation of $CD8^+$ T cell clones. CTL clones were generated by limiting dilution seeding T cells at 0.1, 0.3, 1 or 3 cells per well in U-bottom 96-well plates and cultured in the presence of irradiated feeder cells (allogenic PBMCs and LCLs) using 1 µg/ml phytohemagglutinin (PHA-L) (Sigma-Aldrich) and 150 U/ml of recombinant IL-2. Using this method, the $gp100_{47-52/40-42}$-specific T-cell clone (K631 1C) was successfully isolated and further expanded on irradiated feeder cells. Before being used in an assay, the CTL clone K631 1C was rested for at least 10 days. The CTL clone RG39 specific for the HLA-A*0201-restricted NY-ESO-$1_{157-165}$ [SLLMWITQC] peptide was prepared as described above. $CD8^+$ T cells specific for the HLA-A*02:01-restricted $gp100^{mel}_{209-217}$ [IMDQVPFSV] peptide were obtained by transducing peripheral blood lymphocytes (PBLs) with the $gp100^{mel}$-specific T cell receptor (TCR) as described below. The previously described LG2-37.7.14 and M45-3B $CD8^+$ T cell clones specific for the HLA-A*32:01-restricted $gp100^{mel}_{40-42/47-52}$ and HLA-A*03:01-restricted $gp100^{mel}_{195-202/192}$ spliced peptides, respectively, were expanded on irradiated feeder cells, as described above.

HLA-A03:01-peptide binding affinity. Binding affinity of synthetic peptides were computed with different peptide concentration in a standard binding affinity assay measured by flow cytometry as described elsewhere [4], using HeLa or T2 cells transfected with the HLA-A*03:01-expressing plasmid.

Generation of gp100^{mel}-specific TCR-transduced T cells. Retroviral vectors containing an optimized TCR gene sequence cassette from a HLA-A*02:01-restricted TCR specific for the $_{M210}$gp100$^{mel}_{209\text{-}217}$ epitope were generated [8,9]. The T210M substitution within the gp100$^{mel}_{209\text{-}217}$ sequence was known to increase the immunogenicity of the gp100$^{mel}_{209\text{-}217}$ epitope and the CD8$^+$ T cell response [10]. Variable TCR α and β chain regions were fused to murine constant TCR α and β chain regions and the resulting TCR β chain was linked to the TCR α chain by a P2A element [11]. The TCR transgene cassette was codon-optimized (GeneArt) [12] and cloned into the γ-retroviral vector MP71-PRE using NotI and EcoRI restriction sites. TCR-retrovirus supernatant for the transduction of PBLs was generated as described by transfecting 18 µg TCR vector plasmid into 293T-GALV packaging cells [13]. PBLs from healthy donors were purified using ficoll gradient centrifugation (Biocoll, Biochrom), stimulated in anti-CD3 (5 µg/ml OKT-3, Pharmingen) and anti-CD28 (1 µg/ml, Pharmingen) antibody-coated 24-well plates and transduced with TCR-retrovirus. Transduction efficiency was measured by flow cytometry using an antibody directed against the murine constant TCR β chain (clone H57-597, BioLegend). PBLs were expanded in T cell medium (RPMI1640 + GlutaMax (Gibco, Invitrogen), 10% heat-inactivated FBS (Pan Biotech), 1x penicillin/streptomycin (Gibco), 1x MEM (Gibco) and 1x sodium pyruvate (Gibco)) containing 400 U/ml IL-2 (Chiron Behring). After 13 days of expansion, PBLs were rested for three days in T-cell medium containing 40 U/ml IL-2 and cryopreserved before use in further experiments.

Flow cytometry. Antibodies to human CD8 and IFN-γ were purchased from BD Biosciences. The anti-HLA-A*03:01 monoclonal antibody was purchased by Abcam. Flow cytometry was carried out on a FACScalibur flow cytometer (BD Bioscience) and analyzed using the WinMDI software. To label intracellular antigens including cytokines, cells were first fixed by incubation in 2% paraformaldehyde. After washing, cells were incubated in PBS/1% BSA/0.1% saponin and were labeled with primary antibodies prior to two washes with PBS. To stain for HLA-A*03:01, a FITC-conjugated anti-mouse secondary antibody was added for 20 min before washing and analysis by flow cytometry.

Plasmid construction and transfection. Full-length HLA-A*03:01 cDNA was obtained by RT-PCR from total RNA of HLA-A*03:01$^+$ cells and cloned into pcDNA3/Zeo(+) expression vector (Invitrogen) using the HindIII and XhoI restriction sites. The cDNA for gp100^{mel} encoding the full-length gp100^{mel} protein (isoform 1) was amplified by RT-PCR from the pcDNA3.1/gp100 plasmid and cloned into pcDNA3.1/*myc*-HIS version B (Invitrogen) using EcoRV and XhoI restriction sites to construct a C-terminally *myc*-HIS-tagged gp100. The pcDNA3.1/gp100/*myc*-HIS expression vector was used as a template for site-directed mutagenesis to substitute the threonine residue of gp100 at position 210 into a methionine (T210M) and to generate a pcDNA3.1/gp100^{M210}/*myc**-HIS construct. To create a plasmid

expressing the HA-Ub/gp100$^{mel}_{40-52}$ fusion protein, the ubiquitin open reading frame was amplified from a pEGFPN3/Ubiquitin expression vector (stock laboratory) using a set of appropriate primers (5‘CCAAGCTTATGGAG*TACCCATACGATGTTCCAGATTACGCT*GAGATGCAGATCTTCGTGAAAACCCTT-3` and 5‘-GAGGGATCCACCACCTCTCAGACGCAGGACCAG-3‘) carrying the HA sequence to generate a N-terminally HA-tagged ubiquitin. The HA-Ub PCR product was then cloned into the pcDNA3/Zeo(+) expression vector (Invitrogen). A DNA duplex encoding the gp100$^{mel}_{40-52}$ peptide was constructed using a pair of annealed complementary oligonucleotides, which was subsequently cloned in frame directly at the 3’ end of the HA-Ub coding sequence. The cDNA for CTAG1b encoding the full-length NY-ESO-1 protein was amplified by RT-PCR from total RNA of HT1080 cells and cloned into pcDNA3.1/*myc*-HIS version B (Invitrogen) to construct a C-terminally *myc*-HIS-tagged NY-ESO-1 using the KpnI and XhoI restriction sites. The cDNA of β5i, β1i and β2i subunits (laboratory stock) were cloned as untagged sequences into the pcDNA3.1 expression vector. Correct sequence of all constructs was confirmed by DNA sequence analysis. HeLa or HeLa 33/2 cells were transfected using the Lipofectamine 2000 reagent (Invitrogen) according to the manufacturer’s instructions. T2 cells were transfected using Amaxa® Cell Line Nucleofector® Kit C and the Amaxa Biosystem Nucleofector™ I according to the manufacturer’s instructions.

siRNA transfection. ON-TARGET plus SMART pool of interfering RNA (Pharmacon) were used to knock down Rpn10 (PSMD4, L-011365-00), p97/VCP (L-008727-00), β1i (PSMB9, L-006023-00) and β5i (PSMB8, L-006022-00). ON-TARGET plus non-targeting pool of siRNA with random nucleotides (D-001810-10) was used in each experiment as a negative control. For siRNA transfection, 2×10^5 cells (HeLa, HeLa 33/2 or Mel63a) were plated in a 6-well plate one day before siRNA exposure. After 24 hrs, cells were transfected with 30 nM of each siRNA using the X-tremeGENE siRNA reagent (Roche) following the manufacturer’s instructions.

Antibodies and western blotting. The mouse monoclonal antibodies directed against β5i (clone A-12) and β-actin (clone C4) were obtained from Santa Cruz Biotechnology, Inc. Antibodies directed against proteasome β1 (clone MCP421), β2 (clone MCP165) and α6 (clone MCP20) subunits as well as Rpn10 (clone S5a-18) were purchased from Enzo Life Sciences. The goat anti-β2i polyclonal antibody (PA5-19146) was obtained from Thermo Scientific. Rabbit anti-β5 (ab3330) and anti-β1i (ab3328) antibodies were purchased from Abcam. Other antibodies used in this study include HA.11 (clone 16B12, Covance), gp100^{mel} (ab137078, Abcam) and p97/VCP (MA3-004, Dianova). Cell extracts were prepared using a homogenization buffer (50 mM NaCl, 50 mM Tris (pH 7.5), 5 mM MgCl2, 0.1% NP-40). The homogenate was centrifuged at 14000 rpm for 15 min at 4 °C and the amount of proteins in the supernatant was quantified using a commercially available BCA assay (Pierce). Ten micrograms of the protein extracts were applied to SDS-PAGE and proteins consequently detected by western blot assay.

POD-conjugated secondary antibodies (Calbiochem) were used at a 1:5000 dilution. Bound antibodies were visualized with ECL chemiluminescence (GE Healthcare).

CTL assays. HeLa cells or HeLa 33/2 cells were transiently transfected with HLA-A*03:01, HLA-A*032, HLA-A*0201 together with either $gp100^{mel}$, HA-Ub/$gp100^{mel}_{40-52}$, $_{M210}gp100^{mel}$ or NY-ESO-1 and used as targets cells for their potential to induce the production of IFN-γ by the K631 1C, RG39, LG2-37-7.14, M453B, RG39 CTL clones or $_{M210}gp100^{mel}_{209-217}$-specific TCR-transduced PBLs. Twentyfour hrs after transfection, target cells were serially diluted and then co-cultured with a fixed amount of T cells, resulting in graded effector-to-target (E:T) ratio in a final volume of 100 µl of RPMI 1640 supplemented with 10% FCS in U-bottom 96-well plates. Alternatively, melanoma cells (Ma-Mel15a, Ma-Mel18, Ma-Mel21a or Ma-Mel63a) were used as target cells and their capacity to present the $gp100^{mel}_{47-52/40-42}$ antigenic peptide was assessed as described above. In some experiments, target cells were treated for 2 hrs with epoxomicin (250 nM) before being extensively washed and added to the T cell clones. After overnight incubation, the supernatants were collected and the IFN-γ content was determined using a commercially available human ELISA kit (BD Biosciences) according to the manufacturer’s instructions.

Screening of $gp100_{47-52/40-42}$-specific $CD8^+$ T cells in HLA-A*03:01 healthy donors’ and melanoma patients’ PBMCs. Blood samples from healthy donors and melanoma patients were obtained after informed consent and ethical approval from the Essen tissue depository SCABIO (http://www.uk-essen.de/index.php?id=2961&L=3&id=1809) and from the Universitätsklinikum Erlangen. Patients suffering from metastastic melanoma and treated with $gp100^{mel}_{209-217}$ peptide pulsed dendritic cell vaccination were part of the clinical trials NCT00053391 and NCT00056134 (pre and post therapy samples) at the Universitätsklinikum Erlangen [14]. Donors’ PBMCs were selected based on HLA typing results. The PBMCs were plated at a concentration of 2×10^5 in 96-well plates and cultured with 30 µM of the $gp100^{mel}_{47-52/40-42}$ synthetic peptide in RPMI medium supplemented with 8% human AB serum, 1% penicillin and IL-2 (50 U/ml). After 10 days of culture, the sensitized T cells were tested for their capacity to release IFN-γ upon 16-hr stimulation with the cognate peptide (30 µM) and CD28/CD49d (500 ng/ml) recombinant co-stimulatory molecules (BD Biosciences). Culture supernatants were then collected 16 hrs later and analyzed for their IFN-γ content using a commercially available ELISA kit (BD Biosciences). A negative control without peptide was included in all experiments.

Statistical Analysis

Data were tested for normality distribution and homoscedasticity by Kolmogorov-Smirnov, Shapiro-Wilk and Levene tests. To identify significant difference between groups reported in **Fig. 6**, Kruskal-Wallis followed by Mann-Whitney tests with Bonferroni Post Hoc correction for multiple comparisons were applied. Otherwise, paired and unpaired Student t-test were carried out. Descriptive statistics were carried out with SPSS (version 17) and R; a *p*-value $<= 0.05$ was considered statistically significant.

References.

1 Liepe, J. *et al.* The 20S Proteasome Splicing Activity Discovered by SpliceMet. *PLOS Computational Biology* **6**, e1000830 (2010).

2 Mishto, M. *et al.* Driving Forces of Proteasome-catalyzed Peptide Splicing in Yeast and Humans. *Mol Cell Proteomics* **11**, 1008-1023 (2012).

3 Bellavista, E. *et al.* Immunoproteasome in cancer and neuropathologies: a new therapeutic target? *Curr Pharm Des* **19**, 702-718 (2013).

4 Mishto, M. *et al.* Immunoproteasome LMP2 60HH variant alters MBP epitope generation and reduces the risk to develop multiple sclerosis in Italian female population. *PLoS One* **5**, e9287 (2010).

5 Mishto, M. *et al.* A structural model of 20S immunoproteasomes: effect of LMP2 codon 60 polymorphism on expression, activity, intracellular localisation and insight into the regulatory mechanisms. *Biol Chem* **387**, 417-429 (2006).

6 Mishto, M. *et al.* Proteasome isoforms exhibit only quantitative differences in cleavage and epitope generation. *Eur J Immunol* (2014).

7 Fonteneau, J. F. *et al.* Generation of high quantities of viral and tumor-specific human CD4+ and CD8+ T-cell clones using peptide pulsed mature dendritic cells. *J Immunol Methods* **258**, 111-126 (2001).

8 Sommermeyer, D. *et al.* Designer T cells by T cell receptor replacement. *Eur J Immunol* **36**, 3052-3059 (2006).

9 Morgan, R. A. *et al.* High efficiency TCR gene transfer into primary human lymphocytes affords avid recognition of melanoma tumor antigen glycoprotein 100 and does not alter the recognition of autologous melanoma antigens. *J Immunol* **171**, 3287-3295 (2003).

10 Parkhurst, M. R. *et al.* Improved induction of melanoma-reactive CTL with peptides from the melanoma antigen gp100 modified at HLA-A*0201-binding residues. *J Immunol* **157**, 2539-2548 (1996).

11 Leisegang, M. *et al.* Enhanced functionality of T cell receptor-redirected T cells is defined by the transgene cassette. *J Mol Med (Berl)* **86**, 573-583 (2008).

12 Scholten, K. B. *et al.* Codon modification of T cell receptors allows enhanced functional expression in transgenic human T cells. *Clin Immunol* **119**, 135-145 (2006).

13 Hennig, K. *et al.* HEK293-based production platform for gamma-retroviral (self-inactivating) vectors: application for safe and efficient transfer of COL7A1 cDNA. *Hum Gene Ther Clin Dev* **25**, 218-228 (2014).

14 Baur, A. S. *et al.* Denileukin diftitox (ONTAK) induces a tolerogenic phenotype in dendritic cells and stimulates survival of resting Treg. *Blood* **122**, 2185-2194 (2013).

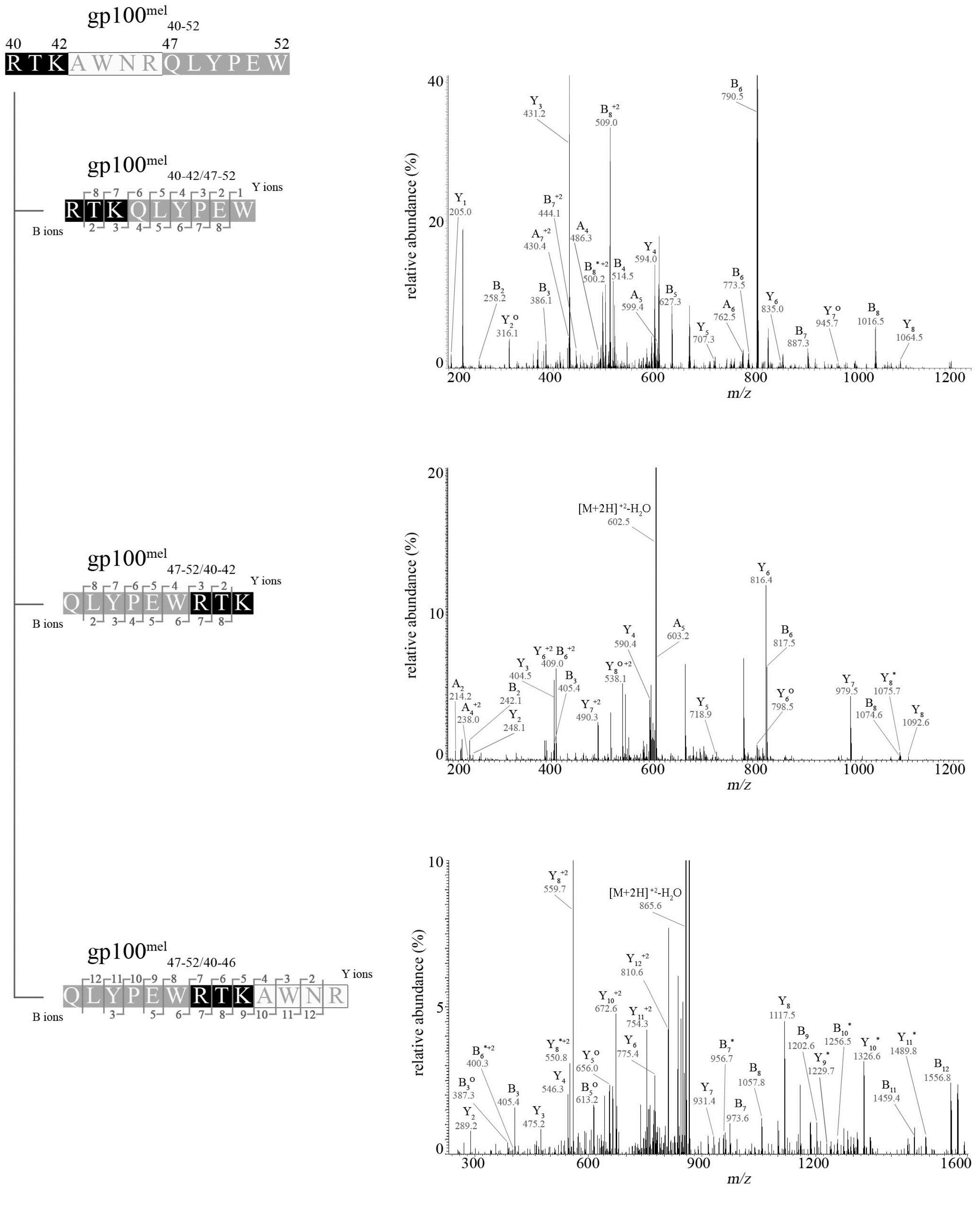

Figure S1. MS/MS identification of proteasome-catalyzed spliced peptides gp100$^{mel}{}_{40\text{-}42/47\text{-}52}$, gp100$^{mel}{}_{47\text{-}52/40\text{-}42}$, gp100$^{mel}{}_{47\text{-}52/40\text{-}46}$ in *in vitro* digestion. Sequence of the synthetic substrate gp100$^{mel}{}_{40\text{-}52}$ and of the spliced peptides gp100$^{mel}{}_{40\text{-}42/47\text{-}52}$ [RTK][QLYPEW], gp100$^{mel}{}_{47\text{-}52/40\text{-}42}$ [QLYPEW][RTK] and gp100-$^{mel}{}_{47\text{-}52/40\text{-}46}$ [QLYPEW][RTKAWNR] identified in the *in vitro* digestion of the synthetic substrate

$gp100^{mel}_{40\text{-}52}$ are depicted. For each spliced peptide the Orbitrap-MS/MS spectrum obtained in the *in vitro* digestion is shown. Detected *m/z* and charges are enclosed in parenthesis. In the spectra single charged B- and Y-ions are reported. Double charged ions are marked as $^{+2}$. In some cases, also prominent A-ions are marked. Ions' loss of water and of ammonia are symbolized by ^{O} and *, respectively. The extracted ion chromatograms of the double protonated spliced peptides identified in the proteasomal reaction showed similar retention time of the corresponding synthetic peptides (data not shown).

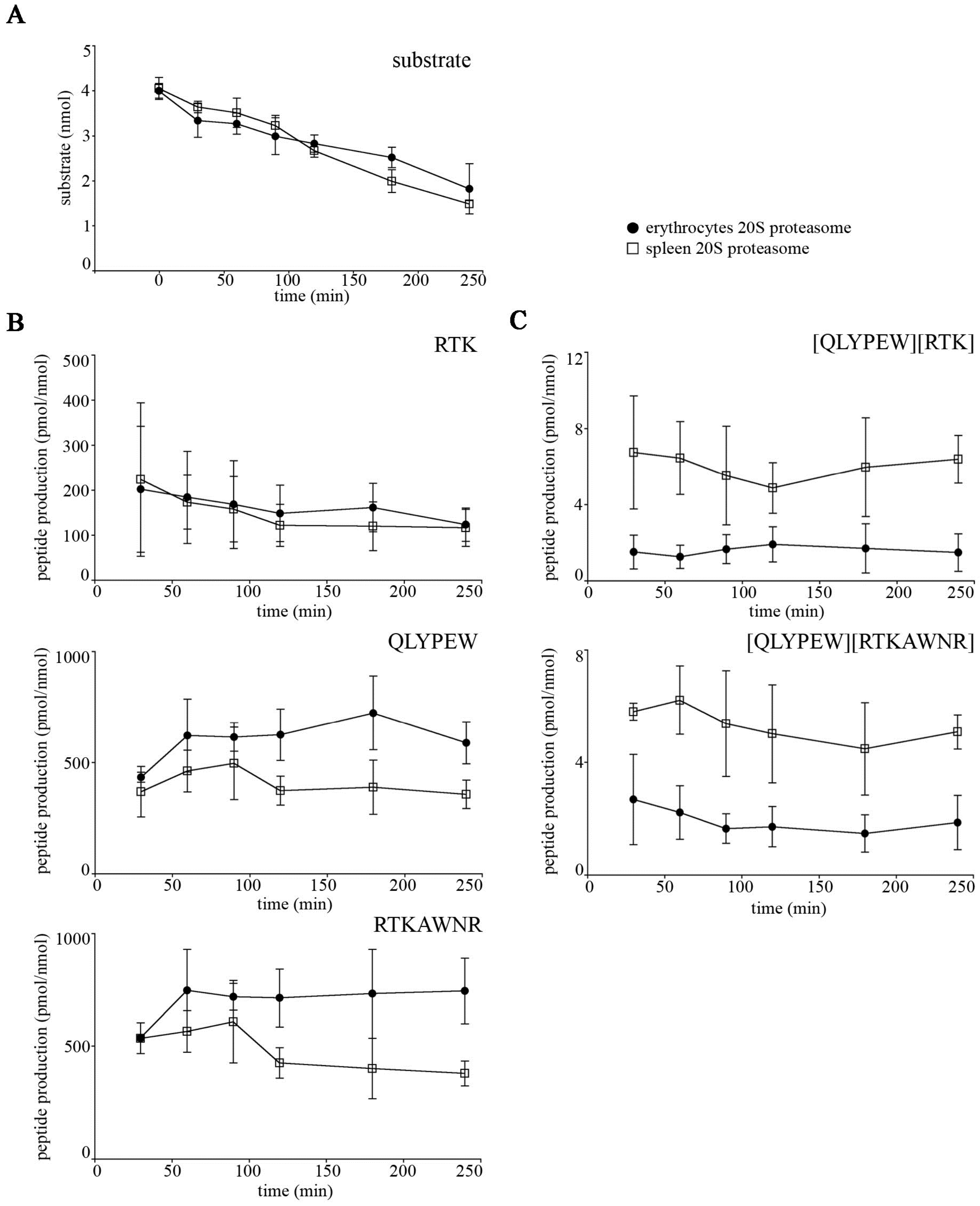

Figure S2. Transpeptidation and condensation efficiency differ from s- to i-proteasome. The PCPS activity of 20S proteasomes purified from human erythrocytes (s-proteasomes) and spleen (mainly i-proteasomes) tissue was monitored by the kinetics of the generation of spliced peptides gp100$^{mel}_{47\text{-}52/40\text{-}42}$ [QLYPEW][RTK] and gp100$^{mel}_{47\text{-}52/40\text{-}46}$ [QLYPEW][RTKAWNR] and of the correlated splice-reactant

peptides during the digestion of the substrate $gp100^{mel}_{40-52}$. Four nmol $gp100^{mel}_{40-52}$ were cleaved in 100 µl reactions by 3 µg of 20S proteasomes for 0 - 4 hours at 37°C. The digestion products were detected by LC-ESI/MS and their absolute amount computed by applying *QME* [2]. Human 20S erythrocyte s- and spleen i-proteasomes revealed a similar $gp100^{mel}_{40-52}$ degradation rate (**A**) as well as efficiency in the generation of the splice-reactant $gp100^{mel}_{40-42}$ [RTK] (**B**). 20S s-proteasome had a larger generation rate of the splice-reactants $gp100^{mel}_{47-52}$ [QLYPEW] and $gp100^{mel}_{40-46}$ [RTKAWNR] than i-proteasome (**B**). (**C**) 20S i-proteasomes exhibited larger production efficiency for the condensation spliced peptides $gp100^{mel}_{47-52/40-42}$ and $gp100^{mel}_{47-52/40-46}$ than 20S s-proteasomes. In (**B, C**) the pmol of peptides produced per nmol of degraded substrate are reported. Bars represent the SD of two independent experiments measured 3 times. A representative time point of this assay is shown in **Fig. 1B-D.**

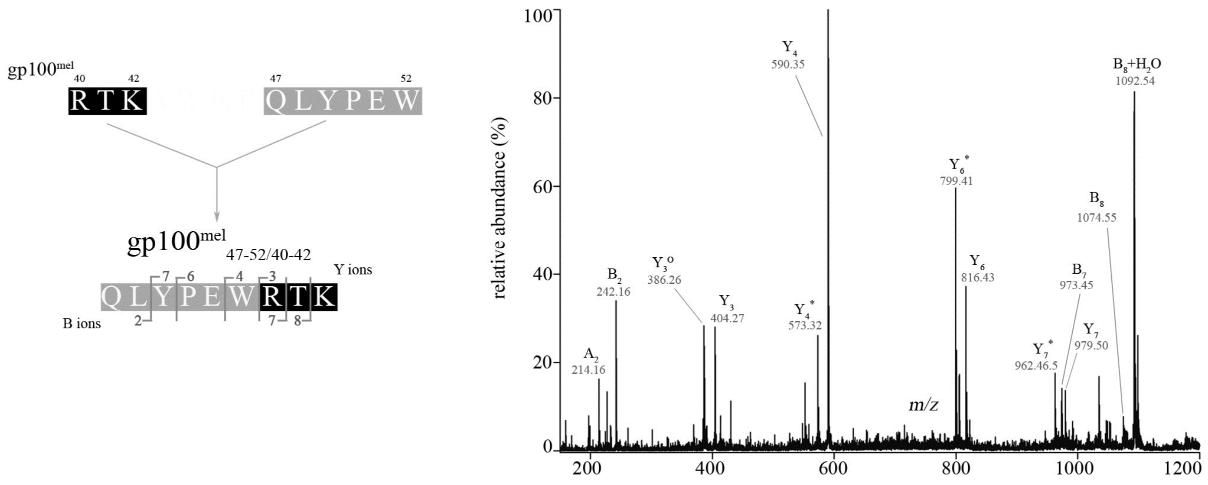

Figure S3. MS identification of the spliced peptide gp100$^{mel}_{47\text{-}52/40\text{-}42}$ in hydrolysis-independent reaction. Sequence of the synthetic splice-reactants gp100$^{mel}_{40\text{-}42}$ and gp100$^{mel}_{47\text{-}52}$ as well as the generated gp100$^{mel}_{47\text{-}52/40\text{-}42}$ spliced epitope identified in the *in vitro* hydrolysis-independent proteasome-catalysed reaction. In the obtained MALDI MS/MS spectrum (precursor mass $[M+H]^+$ = 1220.7) detected *m/z* and charges are enclosed in parenthesis. In the spectra single charged B- and Y-ions are reported. In some cases, also prominent A-ions are marked. Ions' loss of water and of ammonia are symbolized by ^{O} and *, respectively.

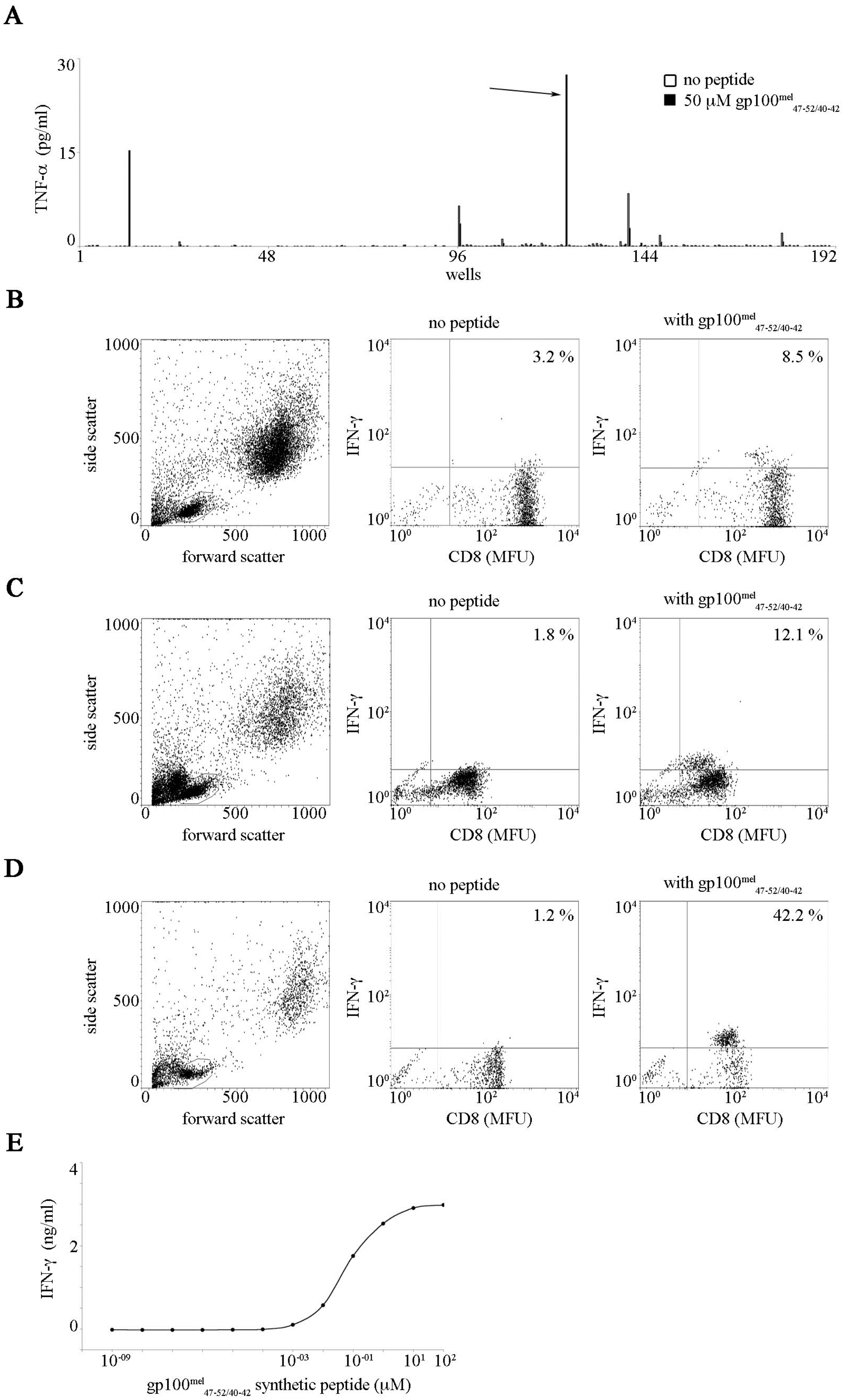

A
TNF-α (pg/ml)
30
15
0
no peptide
50 μM gp100mel 47-52/40-42
wells
1
48
96
144
192
B
no peptide
with gp100mel 47-52/40-42
side scatter
forward scatter
IFN-γ
CD8 (MFU)
3.2 %
8.5 %
C
1.8 %
12.1 %
D
1.2 %
42.2 %
E
IFN-γ (ng/ml)
gp100mel 47-52/40-42 synthetic peptide (μM)

Figure S4. Generation and characterization of the $CD8^+$ T cell clone (K631 1C) specific for the $gp100^{mel}_{47\text{-}52/40\text{-}42}$ spliced epitope. (**A**) $CD8^+$ T cells sensitized with the $gp100^{mel}_{47\text{-}52/40\text{-}42}$ synthetic peptides were analyzed for their capacity to secrete TNF-α upon re-stimulation with 50 µM of the same peptide in a 6 hr assay. A negative control without peptide was included in this experiment. TNF-α was measured using a WEHI assay using TNF-sensitive WEHI cells. The well #136 used in the further isolation and expansion is marked with an arrow. (**B**) The frequency of the $CD8^+$ T cells present in the micro-cultures well #136 was estimated by assessing their production of IFN-γ in response to the peptide by intracellular staining followed by flow cytometry analysis in a 6 hr assay in the presence of 10 µM brefeldin A (BFA). (**C**) $CD8^+$ T cells deriving from the micro-culture #136 were further expanded on U-bottom 96-well plates with feeder cells and IL-2 (150 U/ml) for two weeks. Once the T cells attained their resting state, they were re-stimulated for 6 hrs with 50 µM $gp100^{mel}_{47\text{-}52/40\text{-}42}$ peptide with BFA (10 µM) and double stained for intracellular IFN-γ and CD8 prior to flow cytometry analysis. A negative control without peptide was included in this experiment. (**D**) The expanded $CD8^+$ T cells from the micro-culture #136 were cloned by limiting dilution in the presence of feeder cells and IL-2 (150 U/ml) on 1 cell/well basis using U-bottom 96-well plates. After two weeks of culture, all growing $CD8^+$ T cell clones were tested for their reactivity towards the $gp100^{mel}_{47\text{-}52/40\text{-}42}$ peptide in a 6 hr assay in the presence of BFA (10 µM) by CD8/IFN-γ intracellular double staining followed by flow cytometry analysis. Shown is the CD8/IFN-γ staining upon stimulation with peptide (or not) of the clone K631 1C. (**E**) Recognition of HLA-A*03:01$^+$ HeLa cells pulsed with serial dilutions of the synthetic peptide $gp100^{mel}_{47\text{-}52/40\text{-}42}$ [QLYPEW][RTK] by the K631 1C CTL clone in a 16 hr IFN-γ release assay at a E:T ratio of 1:1. IFN-γ in the supernatants was measured by ELISA.

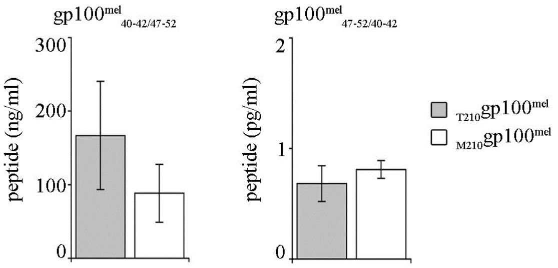

Figure S5. T210M gp100^{mel} substitution does not affect the *in cellulo* presentation of the spliced epitopes gp100$^{mel}_{40-42/47-52}$, gp100$^{mel}_{47-52/40-42}$. CD8^{+} T cell clones specific for the spliced epitopes gp100$^{mel}_{40-42/47-52}$ or gp100$^{mel}_{47-52/40-42}$ were exposed to HLA-A*03:01/-A*02:01/-A*32:01^{+} HeLa cells (E:T = 1:1) transfected with the $_{M210}$gp100^{mel} or the $_{T210}$gp100^{mel}$_{-expressing}$ plasmids. The IFN-γ-release by CD8^{+} T cell clones was compared to a parallel titration assay where CD8^{+} T cell clones were exposed to HLA-A*03:01/-A*02:01/-A*32:01^{+} HeLa cells (E:T = 1:1) pulsed with different concentration of the synthetic peptides. Therefore, we here report a marker of the amount of epitope presented on the cell surface of HLA-A*03:01/-A*02:01/-A*32:01^{+} HeLa cells transfected with the target antigens. By titrations we calculated the amount of synthetic peptide pulsed to HeLa cells corresponding to the IFN-γ released by the specific CD8^{+} T cell clones exposed to HLA-A*03:01/-A*02:01/-A*32:01^{+} HeLa cells transfected with the target antigen and cultivated in an E:T ratio = 4:1 (see also **Fig. 6**). Values are the means and bars the SEM of independent experiments (n = 2; each experiment with three independent replicates). No significant differences in the means were observed (we applied the Mann-Whitney test with Bonferroni correction for multiple comparison). The IFN-γ-release in the medium was measured by ELISA after 16 hrs co-culture.
